# Controllable fabrication of polymeric nanowires by NIL technique and self-assembled AAO template for SERS application

**DOI:** 10.1038/s41598-021-94513-w

**Published:** 2021-07-22

**Authors:** Dongjing Li, Aixia Wu, Qing Wan, Zeping Li

**Affiliations:** School of Electronic Information and Engineering, Hubei University of Science and Technology, Xianning, 437005 People’s Republic of China

**Keywords:** Nanoscale materials, Applied physics

## Abstract

A controllable strategy to fabricate the polymeric nanowires with high throughput and low cost is developed by using the thermal nanoimprint lithography (NIL) technique and self-assembled anodic aluminum oxide (AAO) template. The length of polymeric nanowires can be controlled by adjusting the duration of thermal NIL. A fill mechanism of thermoplastic intermediate polymer stamp (IPS) polymer pressed into the AAO nanopores is closely studied. The as-prepared IPS polymeric nanowire-based Surface-Enhanced Raman Scattering (SERS)-active substrate exhibits a remarkable reproducibility. The effective adsorption of the R6G as probe molecule near to hotspots generated at 3D vertically aligned polymeric nanowire SERS active substrates shows extraordinary enhancement of Raman signal with an enhancement factor (EF) of 10^5^–10^6^. The present strategy is of great guiding significance to broaden the use of thermal NIL technique and AAO template for the fabrication of other nanomaterials, especially for the flexible and transparent polymer-based nanomaterials.

## Introduction

The polymeric nanowires have attracted much attention for their potential applications in the fields of sensor, FET, ECE, Solar Cell, supercapacitor in recent years^1–5^, due to their excellent flexibility, optical and electrical properties. Although polymeric materials are cheap, flexible, transparent, biocompatible, and easy to process^6–8^, how to prepare polymeric nanowires with high throughput and low cost in a controllable way is still a hot topic.

Although the NIL technique offers the possibility for mass production of nanostructures with high resolution at a low cost^9–11^, its initial template has to be fabricated by tedious and time-consuming methods such as electron beam lithography (EBL) or focused ion beam (FIB), which limits its potential applications^12–15^. The AAO membrane has been widely exploited for nano-patterning template due to its the adjustable structural parameters, large patterning area, high throughput and low cost^16–18^.

Flexible SERS-active substrates have advantages over traditional rigid substrates in terms of flexible or wearable tags, such as sensing with non-planar geometry^19^. Due to their excellent mechanical strain resistance, they can be easily customized into desired shape and size^20^. Therefore, while maintaining high sensitivity, SERS substrate with excellent transparency and flexibility may be a potential research area of Raman spectroscopy.

In this paper, we report a controllable strategy for fabricating arrays of polymeric nanowire structures with high throughput and low cost by using the thermal NIL technique and self-assembled AAO template. With the increase of thermal NIL time, the polymeric nanowires gradually formed vertically until they collapsed. And a filling mechanism of thermoplastic IPS pressed into the AAO nanopores was closely studied. Finally, the reproducibility of Raman signals on SERS polymeric nanowire substrate was estimated. And the Raman spectra of R6G molecules on the IPS polymeric nanowire-based SERS-active substrate was measured. The effective adsorption of the R6G as probe molecule near to hotspots generated at 3D vertically aligned polymeric nanowire SERS active substrates showed extraordinary enhancement of Raman signal with an EF of 10^5^–10^6^.

## Results and discussion

### Topography feature of AAO membrane

**Figure 1:**
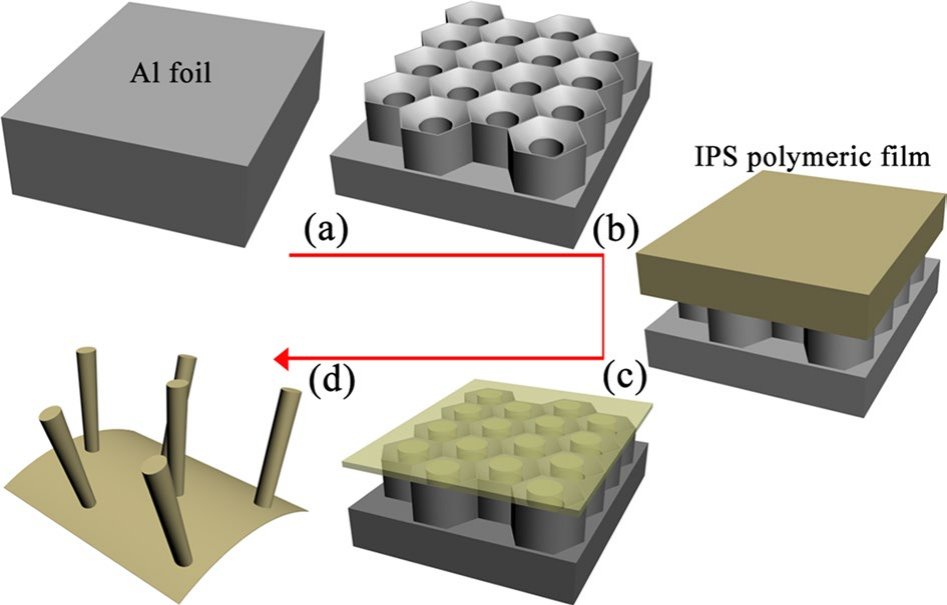
Schematic of fabrication procedure of polymeric nanowire. (**a**) A two-step anodization process, (**b**) Transfer of polymer film onto AAO template after AAO pore-widening and anti-adhesion pretreatment, (**c**) The thermal NIL process. (**d**) Removal of AAO template from polymeric film.

**Figure 2:**
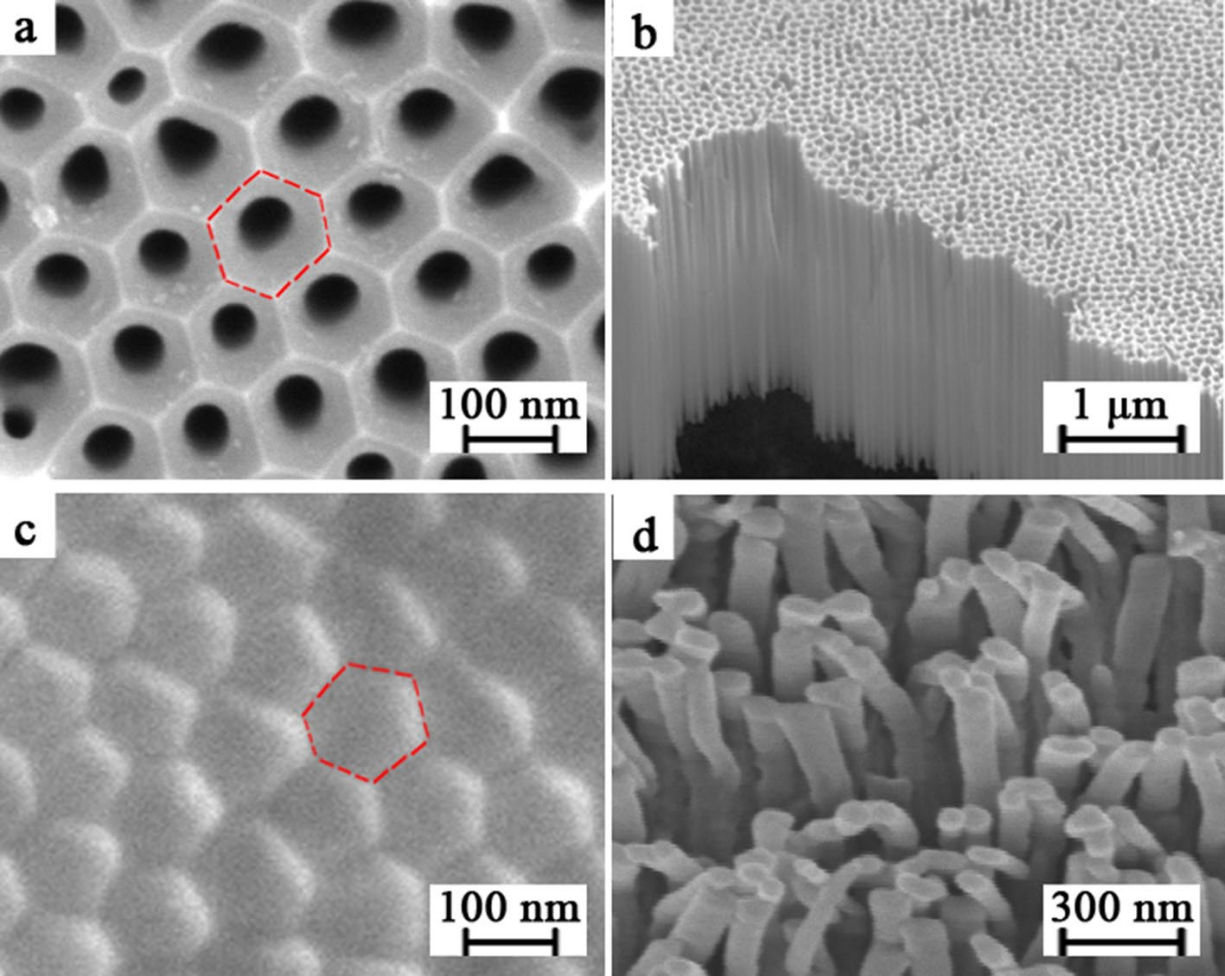
SEM images of samples. (**a**) the surface of AAO membrane before the pore-widening, (**b**) free standing AAO membrane, (**c**) the surface of IPS polymeric for a short duration (3 min) of thermal NIL, and (**d**) the IPS polymeric nanowires for a long duration (40 min) of thermal NIL.

Figure 1 shows the fabrication process of polymeric nanowires (see “Methods” for details). The configurations of the as-prepared AAO membrane and polymeric nanowire are shown in Fig. 2. Under specific anodization conditions, the alumina self-organizes to form a honeycomb-like arrangement, which is a close-packed hexagonal cell array as shown in Fig. 2a,b. The diameter of AAO cells is about 90 nm. Each cylindrical hole and the alumina area around it form a hexagonal cell, which contains a cylindrical central nanohole perpendicular to the surface of the Al foil below as shown in Fig. 2b. The pore diameters within the cell size can be adjusted through pore-widening with acid solutions, and the thickness of the AAO layer can be adjusted by changing the duration of anodization^16–18^. After a short time of thermal NIL (3 Min), the polymer cannot be pressed into the inside of the AAO pore, but on the concave surface of the AAO pore, as shown in Fig. 2a, where the regularity of the AAO surface is inherited by the surface of the polymeric film (see Fig. 2c). But the polymer pressed into the inside of the AAO pore comes to form the polymeric nanowires, under a long duration of thermal NIL (40 Min), as shown in Fig. 2d.

### Controllable fabrication of polymeric nanowires

**Figure 3:**
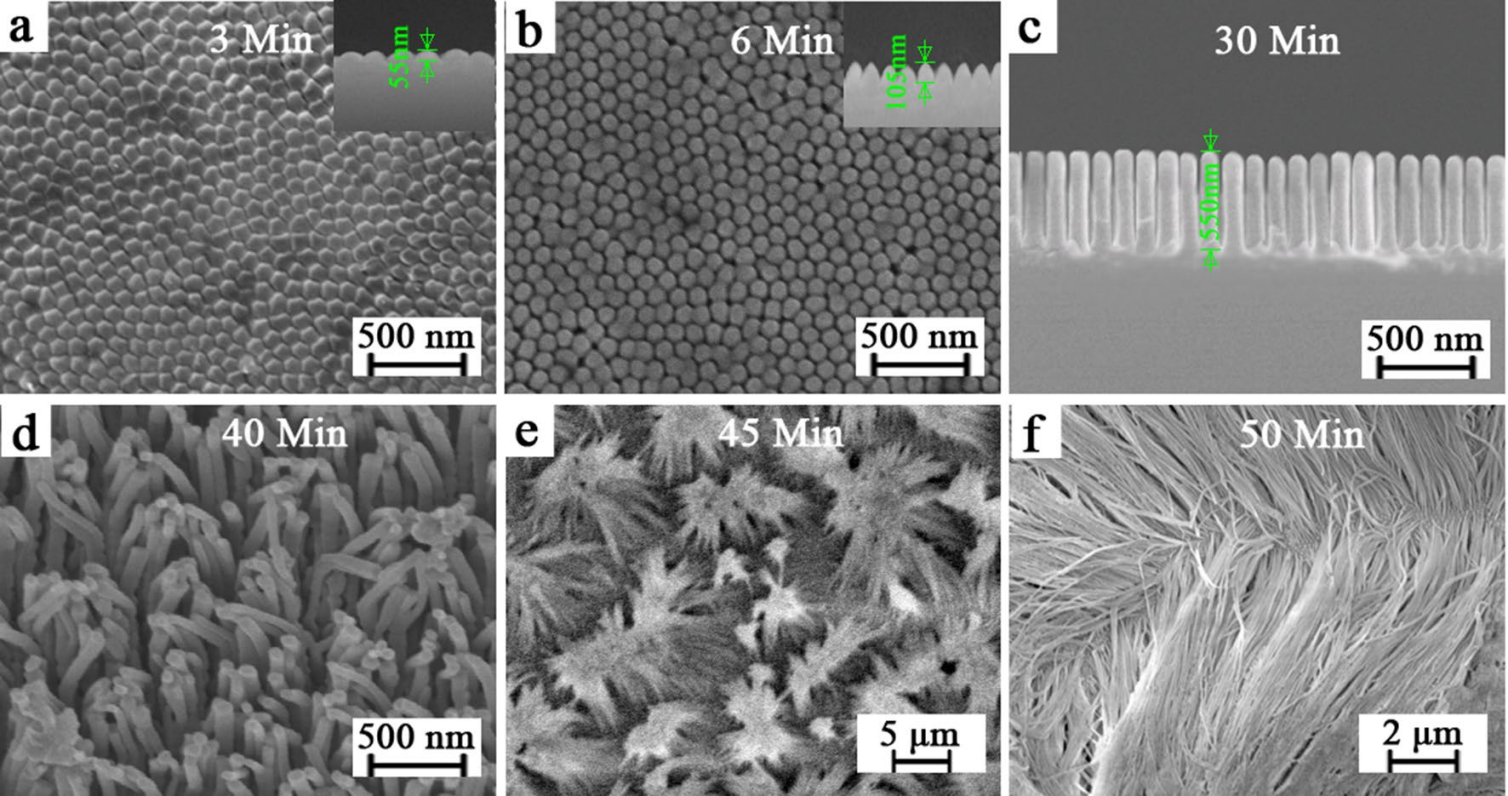
SEM images of IPS from vertically aligned hexagonally patterned IPS polymeric nanowires to collapsed IPS polymeric nanowires. For different (**a**–**f**) duration of thermal NIL, and the insets show cross-sectional view of nanowires.

It is necessary to select appropriate times, the applied pressure, and appropriate temperature of thermal NIL that determine the viscosity of the polymer, and a pressure-assisted template technique was used to ensure that the polymer completely fills the pores of the template during the thermal NIL^24^. In this experiment, the polymeric nanowires (see Fig. 3) were fabricated by pressing a thermoplastic IPS film into the initial AAO template at the temperature of 155 ^◦^C and the pressure of 40 bar respectively for different times. At 3 Min, the polymer cannot be pressed into the inside of the AAO pore, the surface of the polymer inherits the regularity of the AAO surface and formed well as shown in Fig. 3a. When the time of thermal NIL increases to 6 Min, the polymeric nanowires are separated from each other and formed nanowires (see Fig. 3b). As the time of thermal NIL increases to 30 Min, the IPS polymeric nanowires grow significantly (see Fig. 3c), and the diameter of the polymeric nanowire is about 80 nm. The further increase of the length of polymer nanowires leads to polymer nanowires from vertical (see Fig. 3c) to inclined morphology (see Fig. 3d), and finally collapsed, as shown in Fig. 3e,f. These results show that the length of polymeric nanowires can be controlled by adjusting the duration of thermal NIL.

**Figure 4:**
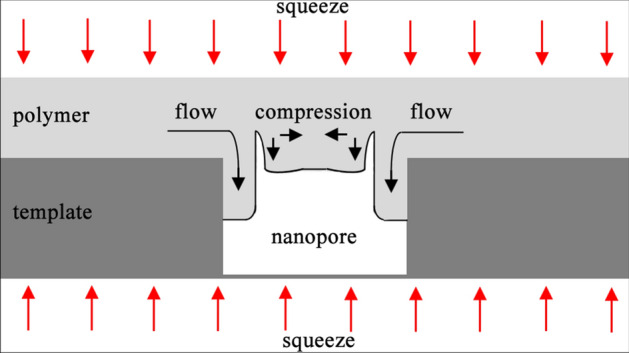
Schematic representation showing polymeric filling of a nanopore during thermal NIL.

A filling mechanism is observed in this thermal NIL process as shown in Fig. 4. The thermoplastic IPS film is shaped by pressing into the AAO template after it is heated above glass transition temperature (T_g_). When the thermoplastic IPS film is compressed between applied pressure and the AAO template, the viscous polymer undergoes a thermal and mechanical compression and is forced to flow into the pores of the AAO, so that it fully conforms to the surface relief of the template. In the case of large-area periodic AAO structures, the specific area which contributes to the filling of a single pore is limited by the structure density or the distances between the pores. The nanostructured region is surrounded by an unstructured area and the majority of material flows from the borders. For this case, the squeeze flow in the interpore regions can be neglected and the effective template area is the area between the edge of the structured region and the template border.

### SERS performance: morphology of the Au-covered polymeric nanowires substrate

**Figure 5:**
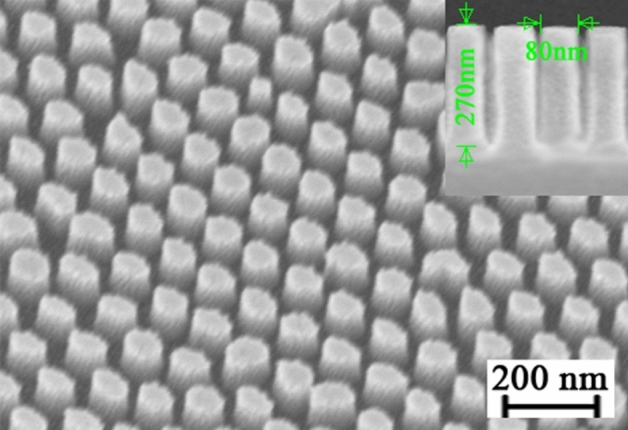
SEM images of the 20 nm Au-coated polymeric nanowire substrate for SERS measurement, and the inset shows cross-sectional view of the nanowires.

Before measurement, 20 nm Au was coated on the vertically aligned polymeric nanowire with the diameter of ~ 80 nm and length of ~ 300 nm under the thermal process time of 20 Min. As shown in Fig. 5, the polymeric nanowires SERS substrate prepared shows a well-ordered distribution. A large number of Au nanoparticles gather on the top of the polymeric nanowires, and a small amount of Au nanoparticles gather on the upper rim of the nanowires by sputtering technology. The hexagonally arranged Au nanoparticles on the polymeric nanowires inherit the periodic hexagonal arrangement of the AAO template, which results in highly uniform shape and size distribution of the coated Au nanoparticles. It is worth mentioning that the vertically aligned polymeric nanowires decorated with Au have an enhanced surface to volume ratio, which will lead to a good enrichment of the analyte molecules. These nanostructures can be used as plasmonic template for surface enhanced Raman scattering (SERS).

### SERS performance: LSPR behavior

**Figure 6:**
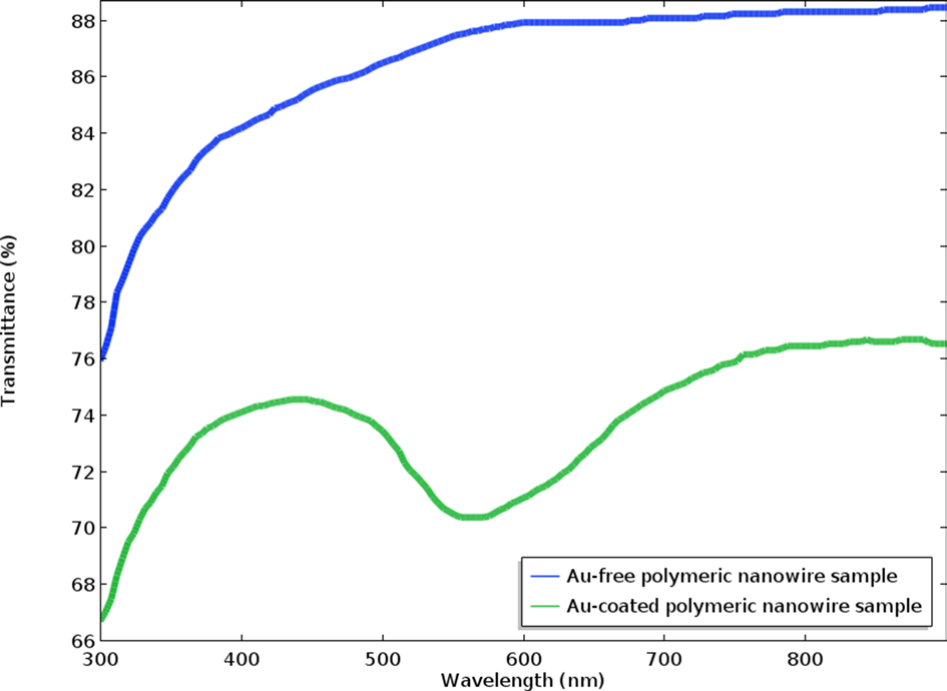
Transmittance spectra showing LSPR spectra of the 20 nm Au-coated polymeric nanowire sample and Au-free polymeric nanowire sample (with the diameter of ~ 80 nm and length of ~ 300 nm).

As well-known, the SERS activity of nanostructured samples strongly depends on their optical properties being related to the strong amplification of the local EM field in the proximity of the nanostructured metallic surface. This amplification is caused by excitation of localized surface plasmonic resonances (LSPR) of individual nanowires or nearby optical coupling structures. Figure 6 shows the transmittance spectra of as-prepared 20 nm Au-coated polymeric nanowire sample and Au-free polymeric nanowire sample as comparison (polymeric nanowire with the diameter of ~ 80 nm and length of ~ 300 nm), in the wavelength range of 300–900 nm. Compared with the Au-free polymeric nanowire sample, the spectrum of the Au-coated polymeric nanowire sample shows a minimum transmittance signal with the center at 550 nm, which can be recognized as a plasmon absorption peak^25^. Such an Au-coated polymeric nanowire sample appear to be optically coupled^25^, leading to the formation of active hot spots, which is then fixed at the excitation wavelength of 550 nm to match the optimal SERS efficiency conditions.

### SERS performance: reproducibility of Raman signals

**Figure 7:**
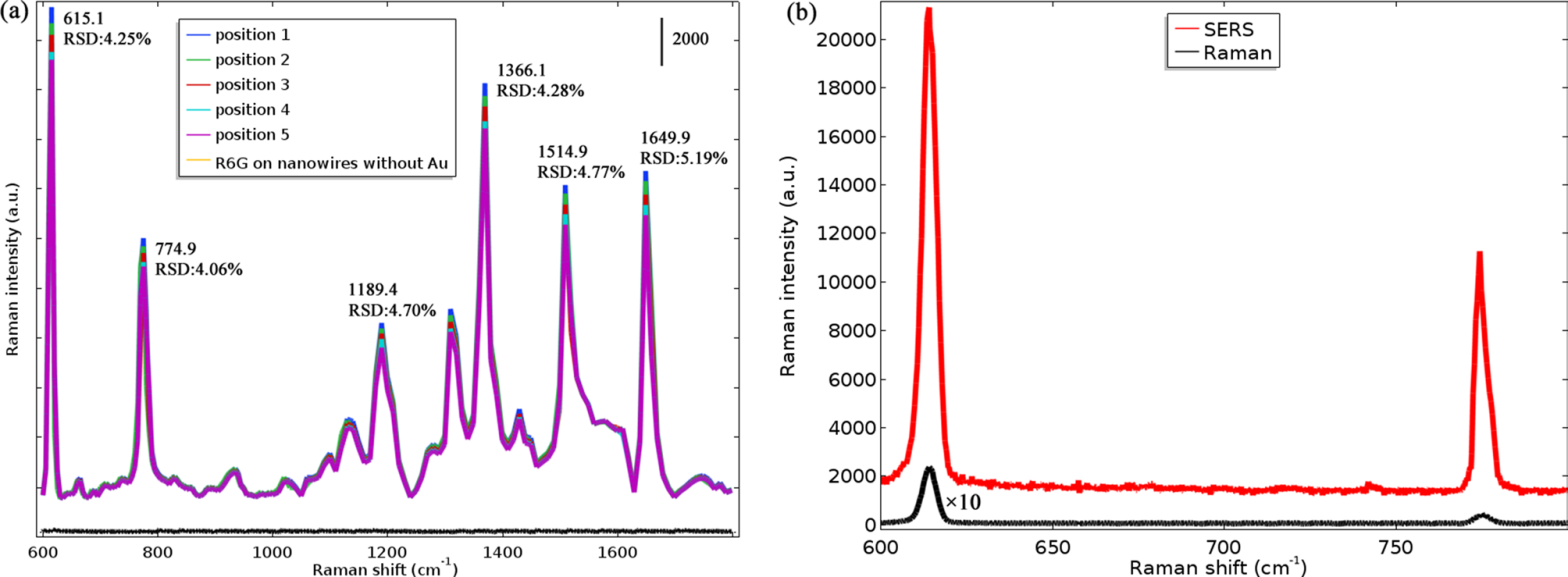
Raman and SERS spectra. (**a**) Measured Raman spectra of R6G (10^−6^ M) molecules illuminated by 532 nm excitation wavelength collected from 5 randomly selected collection points, where RSD is relative standard deviation of Raman intensity at principal Raman peaks. (**b**) Comparison between the 615 cm^−1^ Raman (10^–1^ M R6G) and SERS (10^–6^ M R6G) spectra.

Considering the significance of the reproducibility of Raman signals on SERS substrate in practical applications, we randomly selected five collection points on the as-prepared substrate to collect the SERS spectra of R6G molecules with a concentration of 10^−6^ M from a range of 600 to 1800 cm^−1^ to estimate whether the substrate can provide the reproducible SERS signals of target molecules, as shown in Fig. 7a. Under 532 nm excitation, the Raman signals measured at five different positions of the sample show very similar intensities. The relative standard deviation (RSD) values of Raman intensity at the main Raman peaks are similar, less than 6%. These results elucidate the SERS substrate has good reproducibility of Raman signals throughout the substrate surface, which is beneficial from high uniformity of the substrate. Moreover, there are six principal Raman peaks centered at 615, 775, 1189, 1366, 1515, and 1650 cm^−1^, which is consistent with the characteristic vibrational frequencies of the R6G as a standard Raman probe^26^. In addition, the Raman spectra of R6G on nanowires without Au shows weak Raman signals that is indistinguishable due to the noise, which can be ignored.

### SERS performance: SERS EF calculation

Among these Raman peaks, the maximum enhancement is observed at 615 cm^−1^, which was chosen to estimate the enhancement factor (EF) of SERS substrate according to the method previously reported^27^. According the method described in previous reports^28^, the calculation of the enhancement factor (EF) can be estimated from EF = (I_SERS_/N_SERS_)/(I_Raman_/N_Raman_), where I_SERS_, I_Raman_, N_SERS_ and N_Raman_ are SERS intensity, Raman intensity, the number of probed molecules in SERS and the number of probed molecules in Raman, respectively. The evaluation of the number of probed molecules (N_SERS_ and N_Raman_) is detailed in “Methods”. At the peak of 615 cm^−1^, I _SERS_ and I_Raman_ are about 20,000 and 200, respectively (see Fig. 7b). For the reference sample with R6G at 10^−1^ M on a plane glass substrate, N_Raman_ will be ~ 7.29 × 10^7^. Considering N_SERS_ ≈ 10^4^–10^5^, the enhancement factor (EF) is therefore estimated to be about 10^5^–10^6^. In addition, the R6G absorption band and laser excitation wavelength (532 nm) in the SERS experiment are in resonant conditions, the resulted resonant effect between excitation and analytes molecules absorption gives rise to a resonant Raman effect that provide a further boost to the Raman scattering. Considering the resonant Raman effect, the EF value estimated above is a conservative, lower bound of the real enhancement factor.

## Conclusions

In conclusion, the present research shows that good control of the fabrication of polymeric nanowires can be achieved through a combination of thermal NIL techniques and self-assembled AAO template. The length of polymeric nanowires can be controlled by adjusting the duration of thermal NIL. A fill mechanism of thermoplastic IPS pressed into the AAO nanopores is closely studied. The present method is a facile, cost-effective, reproducible, and low-cost strategy to fabricate SERS polymeric nanowire substrates on a large scale. The as-prepared IPS polymeric nanowire-based SERS-active substrate exhibits a remarkable reproducibility. The effective adsorption of the R6G as probe molecule near to hotspots generated at 3D vertically aligned polymeric nanowire SERS active substrates shows extraordinary enhancement of Raman signal with an EF of 10^5^–10^6^. The present strategy is of great guiding significance to broaden the use of thermal NIL technique and AAO template for the fabrication of other nanomaterials, especially for the flexible and transparent polymer-based nanomaterials.

## Methods

### Synthesis of AAO template

A two-step anodization process is performed for the self-assembled synthesis of the AAO membrane, which results in well-ordered hexagonally arranged nanopore arrays, as shown in Fig. 1a. First, a 0.5 mm thick highly pure (99.99%) aluminum (Al) foil was annealed and cleaned, and then electropolished in a solution of perchloric acid and ethanol (HClO_4_: C_2_H_5_OH = 1:6 in volumetric ratio) at a constant voltage of 20 V for 14 min, to decrease surface roughness and improve grain size. The first anodizing was carried out in 0.3 M oxalic acid solution at 3 °C and a constant voltage of 40 V for 4 h, followed by removal of prepared irregular alumina in a mixture of 1.8 wt % chromic acid and phosphoric acid at 60 °C for 8 h. The second anodizing was performed under the same experimental conditions as the first anodization, in which appropriate times were set to obtain AAO membrane of desired thicknesses. To widen the nanopores, the chemical etching of as-prepared AAO membrane was performed in 5 wt % phosphoric acid solution at 40 °C, in which appropriate etching times were set to obtain the desired diameter of nanopores. The detailed synthesized process has been described in the previous works^21,22^.

### Fabrication of polymeric nanowires

The schematic in Fig. 1 shows the fabrication process of polymeric nanowires. An anti-adhesion pretreatment for the initial NIL template is necessary for the subsequent NIL process. The as-prepared AAO membrane as an initial NIL template immersed in a 0.6 mmol/mL 1H,1H,2H,2H perflurodecyltrichlorosilane (FDTS) /isooctane solution for 10 min to prevent adhesion of the polymeric film to the AAO template^23^. Then it is cleaned with isooctane, acetone, IPA, and water successively, followed by blow-drying. After AAO pore-widening and anti-adhesion pretreatment, the polymeric film is transferred onto AAO template, as shown in Fig. 1b. The polymeric nanowires are fabricated by pressing the relatively soft IPS film ((a thermoplastic polyolefin polymeric film, developed by Obducat AB) against the hard pre-treated AAO template through thermal NIL at the temperature of 155 °C and the pressure of 40 bar respectively as shown in Fig. 1c, in which specific times were set to obtain polymeric nanowires of different length. Finally, when the IPS polymeric nanowires are cooled to a temperature below the glass transition temperature (T_g_), it is obtained after mechanically demolding and residue removal of AAO, as shown in Fig. 1d.

### Characterization

For structural characterization, the surface topography of the AAO membrane and polymeric nanowires is measured using a scanning electron microscope (SEM, FEI, Nova 450). The sputtering technique is used to deposit an Au film with a thickness of 20 nm on the surface of the AAO membrane and polymeric nanowires before taking the SEM images. Before Raman scattering measurements, a drop of 20 μL of 10^−6^ M rhodamine 6G (R6G) molecule aqueous solution was dripped upon top of as-prepared Au-coated polymeric nanowire sample and dried directly on the SERS substrates in an ambient environment. The transmittance spectra were measured with a Perkin Elmer Lambda 35 UV–Vis spectrophotometer. For Raman spectra measurement, a drop of 10 μL of 10^−1^ M R6G solution was dripped on a plane glass substrate. A Raman spectrometer (HR800-UV, Horiba-Jobin Yvon) equipped with a microscope of 100 × objective (NA = 0.9) lens. An Ar + laser (λ = 532 nm) with a power of 5 mW served as the excitation source is used for Raman and SERS measurement. The integration time was 10 s for each spectrum at room temperature. Commercial software (Labspec, Horiba-Jovin Yvon) was used to fit the baseline of all Raman spectra. Raman signals were recorded at five different points of the substrate, and the standard deviation (SD) and relative standard deviation (RSD) of the spectra were calculated.

### SERS enhancement factors: evaluation of the number of probed molecules

According the method described in previous reports^28^, the number of molecules probed in the Raman can be estimated from N_Raman_ = N_Avo_ × c_Raman_ × V_laser_, where N_Avo_ of 6.022 × 10^23^ is the Avogadro number, c_Raman_ is the molar concentration and V_laser_ the diffraction limited volume probed by the microscope objective. V_laser_ can be estimated from V_laser_ = (4π/3) × b_1_b_2_b_3_, where the semi-axes b_1_ = b_2_ = 0.61 × λ/NA, b_3_ = 2 × λ/NA^2^, λ = 633 nm and NA (numerical aperture) = 0.9. Consequently, V_laser_ will be ~ 1.21 μm^3^, N_Raman_ will be ~ 7.29 × 10^8^c_Raman_.

According the method described in previous reports^28^, the number of probed molecules in SERS (N_SERS_) can be calculated from N_SERS_ = N_mol_ × N_nano_, where N_mol_ is the number of molecules surrounding each nanostructure, N_nano_ is the number of nanostructures present in the laser spot. N_mol_ is calculated through the information on the nanowires’ hydrodynamic radius while nanowires are mixed with R6G. The value of N_mol_ is estimated to be the orders of 10^3^. The number of nanowires (N_nano_) in the laser focus can be estimated in the ideal situation where the nanowires totally fill the focal laser spot semi-volume (V_laser_ ≈ 1.21 μ m^3^). The value of N_nano_ is estimated to be the orders of 10^3^, yielding a number of probed molecules N_SERS_ ~ 10^6^. For 80 × 270 nm nanowires, less than 10% of the molecules located and absorbed at edge of the nanowires surface and the hot spots will experience the SERS enhancement, yielding N_SERS_ ≈ 10^4^–10^5^.

## Data Availability

The data used to support the findings of this study are available from the corresponding author upon request.

## References

[1] Song E (2015). Inkjet printing of conductive polymer nanowire network on flexible substrates and its application in chemical sensing. Microelectron. Eng..

[2] Xiao CY (2015). High performance polymer nanowire field-effect transistors with distinct molecular orientations. Adv. Mater..

[3] Zhang GZ (2019). Nanoconfinement-induced giant electrocaloric effect in ferroelectric polymer nanowire array integrated with aluminum oxide membrane to exhibit record cooling power density. Adv. Mater..

[4] Yan H (2014). Doping Poly(3-hexylthiophene) nanowires with selenophene increases the performance of polymer-nanowire solar cells. Chem. Mater..

[5] Wang K (2014). Conducting polymer nanowire arrays for high performance supercapacitors. Small.

[6] Hanif M (2016). Poly(methyl methacrylate) composites with size-selected silver nanoparticles fabricated using cluster beam technique. J. Polym. Sci. Pt. B..

[7] Manohar C (2014). 3D nanostar dimers with a sub-10-nm gap for single-/few-molecule surface-enhanced Raman scattering. Adv. Mater..

[8] Hasell T (2008). Silver nanoparticle impregnated polycarbonate substrates for surface enhanced Raman spectroscopy. Adv. Funct. Mater..

[9] Sun KX (2013). Gap-tunable Ag-nanorod arrays on alumina nanotip arrays as effective SERS substrates. J. Mater. Chem. C.

[10] Ou FS (2011). Hot-spot engineering in polygonal nanofinger assemblies for surface enhanced Raman spectroscopy. Nano Lett..

[11] Barcelo SJ (2012). Fabrication of determ**i**nistic nanostructure assemblies with sub-nanometer spacing using a nanoimprinting transfer technique. ACS Nano.

[12] Guo LJ (2007). Nanoimprint lithography: Methods and material requirements. Adv. Mater..

[13] Torres CMS (2003). Nanoimprint lithography: An alternative nanofabrication approach. Mater. Sci. Eng. C.

[14] Wu C (2012). Anti-adhesion treatment for nanoimprint stamps using atmospheric pressure plasma CVD (APPCVD). Appl. Surf. Sci..

[15] Liu QJ (2021). On-chip experiment for chiral mode transfer without enclosing an exceptional point. Phys. Rev. A.

[16] Keller F (1953). Structural features of oxide coatings on aluminum. J. Electrochem. Soc..

[17] Lee W (2014). Porous anodic aluminum oxide: Anodization and templated synthesis of functional nanostructures. Chem. Rev..

[18] Lei Y (2005). Highly ordered arrays of metal/semiconductor core−shell nanoparticles with tunable nanostructures and photoluminescence. J. Am. Chem. Soc..

[19] Singh JP (2012). Flexible and mechanical strain resistant large area SERS active substrates. Nanoscale.

[20] He D (2009). Large-scale synthesis of flexible free-standing SERS substrates with high sensitivity: Electrospun PVA nanofibers embedded with controlled alignment of silver nanoparticles. ACS Nano.

[21] Li ZP (2017). Fabrication of nanopore and nanoparticle arrays with high aspect ratio AAO masks. Nanotechnology.

[22] Li ZP (2017). Pattern transfer of hexagonal packed structure via ultrathin metal nanomesh masks for formation of Si nanopore arrays. J. Alloy Compd..

[23] Beck M (2002). Improving stamps for 10 nm level wafer scale nanoimprint lithography. Microelectron. Eng..

[24] Koo N (2008). The fabrication of a flexible mold for high resolution soft ultraviolet nanoimprint lithography. Nanotechnology.

[25] Romero I (2006). Plasmons in nearly touching metallic nanoparticles: Singular response in the limit of touching dimers. Opt. Express.

[26] Xue TY (2016). R6G molecule induced modulation of the optical properties of reduced graphene oxide nanosheets for use in ultrasensitive SPR sensing. Sci. Rep..

[27] Lim DK (2009). Nanogap-engineerable Raman-active nanodumbbells for single-molecule detection. Nat. Mater..

[28] Fazio B (2016). SERS detection of biomolecules at physiological pH via aggregation of gold nanorods mediated by optical forces and plasmonic heating. Sci. Rep..

